# Yerba Mate as a Source of Elements and Bioactive Compounds with Antioxidant Activity

**DOI:** 10.3390/antiox11020371

**Published:** 2022-02-12

**Authors:** Elżbieta Rząsa-Duran, Agata Kryczyk-Poprawa, Dawid Drabicki, Adrian Podkowa, Katarzyna Sułkowska-Ziaja, Agnieszka Szewczyk, Katarzyna Kała, Włodzimierz Opoka, Piotr Zięba, Maciej Fidurski, Bożena Muszyńska

**Affiliations:** 1 Branch in Kraków—Hospital Pharmacy, Maria Sklodowska-Curie National Research Institute of Oncology, Garncarska 11 Str., 31-115 Krakow, Poland; duranela@poczta.onet.pl; 2 Department of Inorganic and Analytical Chemistry, Faculty of Pharmacy, Jagiellonian University Medical College, Medyczna 9 Str., 30-688 Krakow, Poland; dawid.drabicki@student.uj.edu.pl (D.D.); adrian.podkowa@uj.edu.pl (A.P.); wlodzimierz.opoka@uj.edu.pl (W.O.); 3 Department of Pharmaceutical Botany, Faculty of Pharmacy, Jagiellonian University Medical College, Medyczna 9 Str., 30-688 Krakow, Poland; katarzyna.sulkowska-ziaja@uj.edu.pl (K.S.-Z.); agnieszka.szewczyk@uj.edu.pl (A.S.); k.kala@uj.edu.pl (K.K.); 4 Department of Horticulture, Faculty of Biotechnology and Horticulture, University of Agriculture in Kraków, 29 Listopada 54, 31-425 Krakow, Poland; p.zieba90@gmail.com (P.Z.); maciej.fidurski@urk.edu.pl (M.F.)

**Keywords:** *Ilex paraguariensis*, yerba mate, antioxidants, bioelements, production process, roasted yerba mate

## Abstract

*Ilex paraguariensis* (yerba mate) is a plant species of the holly genus Ilex native to South America from the family Aquifoliaceae and is used for the production of yerba mate infusion. The leaves of the plant are steeped in hot water to make a beverage known as mate. The present study aimed to quantify and compare the content of selected elements in dried leaves and stems of *I. paraguariensis* (originating from Paraguay, Argentina, and Brazil) available in the market in Poland and determine the amount of these elements and bioactive compounds that pass into the infusion prepared from them. The contents of the following antioxidant compounds were assessed: neochlorogenic acid, chlorogenic acid, cryptochlorogenic acid, caffeic acid, 4-feruloylquinic acid, isochlorogenic acid, rutoside, astragalin, caffeine, and indole derivatives. All the tested samples showed the presence of elements such as magnesium, zinc, copper, iron, and manganese. The highest antioxidant activity was determined for infusion prepared from yerba mate samples from Brazil. Drinking approximately 1 L of the infusion a day will partially cover the daily requirement of these elements and bioactive compounds. The highest content of organic compounds with antioxidant properties (phenolic compounds and caffeine) was found in yerba mate infusions from Brazil.

## 1. Introduction

*Ilex paraguariensis* A. St.-Hil. (ang. Paraguay holly, yerba mate) is a plant species of the holly genus Ilex native to South America from the family Aquifoliaceae and is used for the production of yerba mate tea (*Mate folium*). Mate was first consumed by the indigenous Guaraní people, and the practice of Mate consumption then spread to the Tupí people, who lived in the department of Amambay and the Alto Paraná territory of Paraguay. The plant was also primarily found in the southern regions of Brazil, Argentina, and Uruguay. In the mid-17th century, the Jesuits managed to domesticate the plant and establish plantations. It is also a medicinal plant known since pre-Columbian times and was used by the Indians to reduce the feeling of hunger, fatigue, and stress; as a diuretic; and as a physical and mental stimulant [1,2,3,4].

Mate products also have the property of detoxification and possess precognitive, hypocholesterolemic, anticancer, and slimming activities. The presence of a large number of chemical compounds and elements in yerba mate indicates the potential antioxidant properties of the raw material.

The leaves of the plant are steeped in hot water to make a beverage known as Mate. Both the plant leaves and the beverage contain purine alkaloids, such as caffeine, and a variety of polyphenols, such as the flavonoids quercetin and rutin, saponins, vitamins, and bioelements. These substances are responsible for the bioactivity of Mate [5,6,7,8,9,10,11,12,13]. 

*I. paraguariensis* infusions show protective activity against the oxidation of low-density lipoprotein by free radicals. It also reduces the number of products formed in the process of lipid peroxidation and increases the level of antioxidants in human blood serum. [14] In vivo studies have demonstrated that the aqueous extract of yerba mate shows a potent anti-obesity effect by modulating the expression of several obesity-related genes. Positive effects of Mate inhibiting the proliferation of colon (HT-29), esophageal, and bladder cancer cells have also been found [1,14,15]. Moreover, *I. paraguariensis* infusions have anti-inflammatory effects, as they inhibit the production of nitric oxide, prostaglandin 2, interleukin-6, and interleukin-1ß [16]. Mate is, however, most often consumed for its stimulating properties. yerba mate extract, when consumed in quantities not exceeding 2.5 L/day, has proven health-promoting effects. A study conducted in Uruguay showed an association between the consumption of more than 2.5 L/day of hot aqueous yerba mate extract and an increased risk of esophageal cancer [17].

The name “yerba mate” originated in Spain. The word “yerba” means a drink made from the herb, while the word “mate” means drinking from a calabash mate gourd. The raw material is pre-dried directly over the hearth at temperatures ranging from 250 °C to 550°C for several seconds or several minutes. The leaves are then dried until they reach a moisture content of 3–6%, which usually takes 8 to 24 h. The dried leaves are then subjected to an aging process that can last up to several months. The aging process produces the distinctive flavor of the *I. paraguariensis* leaves. In the next stage, the obtained dried product is grounded and packed. The conditions of all the abovementioned steps have a significant impact on the taste, aroma, quality, and contents of biologically active substances in the final product [1,4].

Yerba mate can be brewed in several ways that vary from country to country or region to region. The leaves of *I. paraguariensis* are brewed differently in Argentina and Brazil, and Paraguay and Uruguay also follow different methods to brew the leaves. The simplest method of preparing an infusion involves placing a bombilla in a container, adding dried leaves of *I. paraguariensis* to fill one-half to three-quarters of the container, and pouring hot, but not boiling, water. Ideally, the water temperature should be around 85–95 °C [18]. Infusions prepared from pure dried holly leaves are usually greenish or greenish-brown in color and have a bitter taste. This taste is very specific to the infusion and is both liked and disliked by consumers. Presently, to improve or mask the bitter taste, several producers add various kinds of fruits, flowers, or herbs to the brewed infusion. Sometimes, such additives not only change the taste of the brew but also enhance its properties; for example, the addition of grounded guarana (*Paullinia cupana*) leaves increases the stimulating properties of yerba mate as the caffeine content of the brew increases.

Because of the increasing popularity of yerba mate, the present study aimed to quantify and compare the content of selected bioelements and bioactive compounds (phenolic compounds, caffeine, and indole derivatives) in dried *I. paraguariensis* leaves and stems (originating from Paraguay, Argentina, and Brazil) available in the market in Poland. The contents of elements such as magnesium (Mg), copper (Cu), iron (Fe), zinc (Zn), and manganese (Mn) in the dried leaves of *I. paraguariensis* were determined, and the amount of these bioelements extracted into the infusion was then assessed. The content of organic compounds in the prepared infusions was also evaluated. Next, the extent to which the daily requirement for Mg, Zn, and Mn is represented by the extracted elemental ions and phenolic compounds and caffeine was determined. Nine yerba mate samples from different manufacturers were used in the study. Three were from an Argentine crop, two from a Paraguayan crop, two from a Brazilian crop, and two from a Brazilian crop destined for the Uruguayan market.

## 2. Materials and Methods

### 2.1. Reagents and Materials

Suprapur^®^ nitric acid (65%) and Suprapur^®^ hydrogen peroxide (30%) were purchased from Merck (Darmstadt, Germany). Quadruple-distilled water with a conductivity of less than 1 µS/cm was obtained using an S2–97A2 distillation apparatus (Chemland, Stargard Szczecinski, Poland). Standards of Zn(II), Fe(III), Mn(II), Mg(II), and Cu(II) at concentrations of 1 g/L were purchased from the District Office of Measures in Łodz, Poland.

The reagents used for the analysis of phenolic acids in the samples were as follows: MeOH and glacial acetic acid of analytical grade were purchased from Chempur, and MeOH of HPLC grade was purchased from Merck. The following standards were purchased: caffeic acid, chlorogenic acid, neochlorogenic acid, and rutoside (Sigma Aldrich, St. Louis, MI, USA); cryptochlorogenic acid, isochlorogenic acid, 4-feruloylquinic acid, and astragalin (ChromaDex, Los Angeles, CA, USA); and caffeine (Fluka Chemie AG, Buchs, Switzerland).

### 2.2. Analysis of Yerba Mate Materials

**Figure 1:**
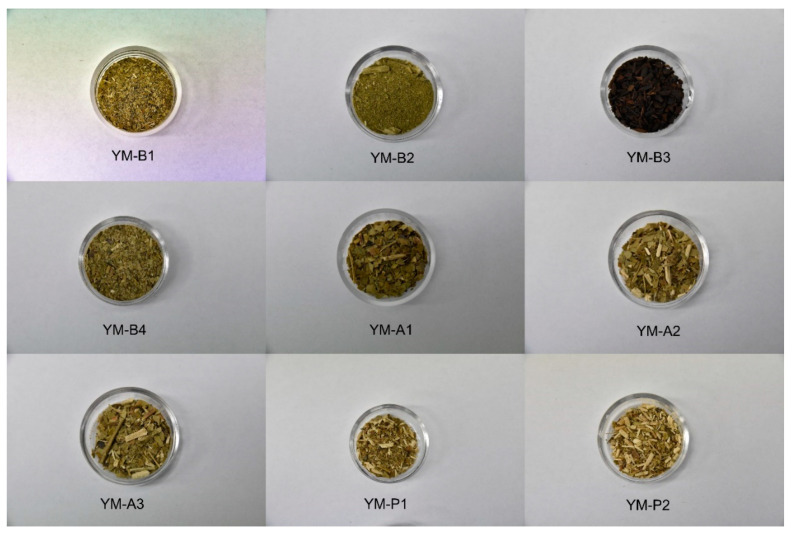
Analyzed products of yerba mate.

The dried leaves and stems of *I. paraguariensis* were purchased from a commercial origin (Amanda, Posada, Argentina; Molimos, Buenos Aires, Argentina; Aquantadora, Misiones, Argentina; Organico, Barao de Cotegipe, Brazil; Beldo, Encantado, Brazil; SAB, Barao de Cotegipe, Brazil; Agro Ventas, Asuncion, Paraguay; Lauroraatz, Itapua, Paraguay). These samples were obtained from different manufacturers and differed in their country of origin. The analysis included yerba mate samples from 9 producers whose names were encrypted. The samples of materials under study were deposited in the Department of Inorganic and Analytical Chemistry Jagiellonian University Medical College. Figure 1 shows samples of the analyzed yerba mate products. The selected products of yerba mate are described as follows: YM-B1—country of origin: Brazil; YM-B2—country of origin: Brazil; YM-B3—country of origin: Brazil (raw material in the product after thermal treatment—roasting); YM-B4—country of origin: Brazil; YM-A1—country of origin: Argentina; YM-A2—country of origin: Argentina; YM-A3—country of origin: Argentina; YM-P1—country of origin: Paraguay; YM-P2—country of origin: Paraguay.

### 2.3. Samples

Dried leaves and stems of *I. paraguariensis* were homogenized, and the samples were weighed to ~0.5000 g with 0.1 mg accuracy in a Teflon vessel. Next, 5 mL of concentrated HNO_3_ solution (65%) and 2 mL of H_2_O_2_ solution (30%) were added. The sample was mineralized in a Magnum II microwave apparatus (ERTEC, Wrocław, Poland) for one cycle of 30 min at a power of 100%, a pressure range of 42–45 Ba, and a temperature range of 250–300 °C. After mineralization, the solutions were transferred to quartz evaporators and evaporated on a heating plate at 150 °C to remove excess acid and form a semidry mass or the so-called “almost dry” stage. The residue obtained after evaporation was transferred to 10 mL volumetric flasks, and it was then diluted up to the mark with fourfold distilled water with a conductivity of <1 μS/cm. The sample solutions were refrigerated until the determination of the metals.

### 2.4. Preparation of Yerba Mate Infusion

The infusions of yerba mate were prepared as follows. Approximately 4.0 g of the dried material from each sample was weighed, 60 mL of water at 85–95 °C was added to the sample, and the solution was then filtered two times after brewing (20 min) and frozen until analysis. The content of bioactive compounds and natural antioxidants was analyzed in all infusions. Furthermore, the content of elements was determined only in infusion with the highest content of Mg^2+^, Zn^2+^, and Mn^2+^ and in those with the lowest values.

### 2.5. Determination of the Tested Elements by Flame Atomic Absorption Spectroscopy

Standard solutions of the tested elements of appropriate concentrations were prepared by serial dilution of the standards with distilled fourfold water. The glassware used to test the elemental content was washed with concentrated nitric acid and distilled water. The content of the investigated elements was determined by flame atomic absorbtion spectroscopy (F-AAS) using an iCE 3000 Series atomic absorption spectrometer (UK) equipped with a flame atomizer and SOLAAR SOLAAR SOFTWARE iCE3000 Series, Version 2.01 software (Thermo Fisher Scientific, Waltham, MA, USA). The quality of the performed analyses was tested using a certified reference material: tobacco leaves Oriental (CTA-VTL-2, Institute of Chemistry and Nuclear Technology, Warsaw, Poland). All results were consistent with certified elemental concentrations. Confirmation of quality control and validation of the methodology used have been previously described using the same methodology and apparatus [19,20]. The obtained concentration was calculated on the basis of the mass of the weighed amounts and solutions along with the mean mass of the samples.

### 2.6. Determination of Organic Compounds in Yerba Mate Products

To determine the content of phenolic acids, flavonoids, and caffeine in the tested materials, 5 g of samples was extracted with methanol for 30 min in an ultrasonic bath at a frequency of 49 kHz (POLSONIC 2, Warsaw, Poland). The extraction process was repeated three times for each sample. The obtained extracts were concentrated by evaporation drying in a rotating vacuum oven at 22 ± 2 °C. The extracts were dissolved quantitatively in HPLC-grade methanol and re-filtered through membrane filters. The prepared methanol extracts were used for determining the content of phenolic acids using RP-HPLC according to an optimized procedure described elsewhere [21]. The process was performed using an HPLC (Merck-Hitachi, Merck KGaA, Darmstadt, Germany) apparatus equipped with the following: an L-2200 autosampler, an L-2130 pump, a LiChrospher RP-18e column (250 mm × 4 mm, 5 µm) thermostated at 25 °C, an L-2350 column oven, and an L-2455 diode array detector at a UV range of 200–400 nm. The mobile phase comprised methanol (solvent A) and methanol/0.5% acetic acid (solvent B) (1:4, *v/v*). The phenolic compounds were quantitatively analyzed using a calibration curve with the assumption of the linear size of the area under the peak and the concentration of the reference standard.

### 2.7. Determination of Indole Derivatives

The HPLC method for determining indole derivatives was performed according to the procedure described by Muszyńska [22]. Briefly, the conditions were as follows: Hitachi HPLC; pump L-7100; column Purospher RP-18 (250 mm × 4 mm, 5 µm). Isocratic separation was used, and the mobile phase was methanol: water: ammonium acetate (15:14:1, *v/v/v*) at a flow rate of 1 mL/min. Chromatographic peaks were recorded at a wavelength of 280 nm. Indole standards were purchased from Sigma (St. Louis, MI, USA).

### 2.8. Determination of Antioxidant Activity Using DPPH Method 

DPPH antioxidant activity was tested using the method described by Molyneux [23], with the DPPH radical (2,2-diphenyl-1-picrylhydrazyl). A quantity of 0.1 mL prepared yerba mate infusion was dissolved in 2.9 mL 80% methanol and mingled. The solution (0.1 mL) was mixed with 4.9 mL of 0.1 mM DPPH^•^ dissolved in 80% methanol. The reaction mixture was shaken, then incubated in the dark at room temperature for 15 min. The absorbance of the solution was measured at 517 nm against the blank using a UV/VIS Helios Beta spectrophotometer. The antioxidant activity was calculated as DPPH [%] = [(A0 − A1)/A0] × 100, where A0 and A1 are the absorbance of the reference and test solutions, respectively.

### 2.9. Determination of Total Phenolic Compounds (TPC)

Phenolic compounds were determined using the Folin–Ciocalteu method described by Djeridane et al. [24]. A quantity of 0.1 mL prepared yerba mate infusion was dissolved in 2.9 mL 80% methanol and mingled. The solution (0.1 mL) was mixed with 2 mL of sodium carbonate; then, after 2 min, Folin–Ciocalteu’s reagent (0.1 mL) mixed with deionized water (1:1 *v/v*) was added to the test tubes. The reaction mixture was incubated at room temperature in the dark for 30 min. The absorbance was measured at 750 nm using the UV–VIS Helios Beta spectrophotometer (Thermo Fisher Scientific Inc., Waltham, MA, USA). The total phenolic content was calculated on the basis of the calibration curve of gallic acid and expressed as the gallic acid equivalents per 1 mL of yerba mate infusion.

### 2.10. Determination of Total Flavonoid Compounds (TFC)

Total flavonoids were determined by the aluminum chloride method using quercetin as a standard [25]. A quantity of 0.1 mL prepared yerba mate infusion was dissolved in 2.9 mL 80% methanol and mingled. Then, 1 mL of the solution was added to a 10 mL volumetric flask containing 1.4 mL of methanol acidified with acetic acid 5% (*v/v*). A quantity of 0.1 mL 2% AlCl3 was added to the flask. After 30 min, the solution was mixed, and the absorbance was measured against the prepared reagent blank at 415 nm using the UV–VIS Helios Beta spectrophotometer (Thermo Fisher Scientific Inc., Waltham, MA, USA). The total flavonoids in the sample were expressed as mg of quercetin equivalents per 1 mL of yerba mate infusion.

### 2.11. Determination of Antioxidant Activity Using FRAP Method

FRAP antioxidant activity was tested using the method described by Benzie and Strain [26]. The FRAP reagent contained 2.5 mL of a 10 mmol/L TPTZ (2,4,6-tripyridyl-s-triazine) solution in 40 mmol/L HCl plus 2.5 mL of 20 mmol/L FeCl_3_, and 25 mL of 30 mmol/L acetate buffer, pH 3.6, was prepared. A quantity of 0.1 mL prepared yerba mate infusion was dissolved in 4.9 mL distilled water and mingled. Then, 0.1 mL of the solution was mixed with 2 mL acetate buffer, and 0.1 mL FRAP reagent was added with 0.1 mL FeCl_3_ solution. The absorbance of the reaction mixture at 593 nm was measured using the UV–VIS Helios Beta spectrophotometer after 15 min and 30 min. Solutions with known Fe (II) concentrations were used to prepare the calibration curve. FRAP results were expressed as µM Fe^2+^ per 1 mL of Yerba Mate infusion.

### 2.12. Statistical Analysis

All samples were prepared and analyzed in triplicate. The results are expressed as means ± standard deviation (SD) in percentages. Data were analyzed by Microsoft Office Excel 365 for Windows. Statistically significant differences were analyzed using the Kruskal–Wallis test with Dunn’s post hoc test. Statistical significance was set at *p* < 0.05 (GraphPad InStat, San Diego, USA). For colorimetric analysis (1.8–1.11) Tukey’s test was performed using the Statistica 13.3 package (TIBCO Software Inc., Palo Alto, CA, USA), and *p*-values of less than or equal to 0.05 were considered to be statistically significant.

## 3. Results and Discussion

### 3.1. Selected Elements Determined in Yerba Mate by Atomic Absorption Spectrometry

**Figure 2:**
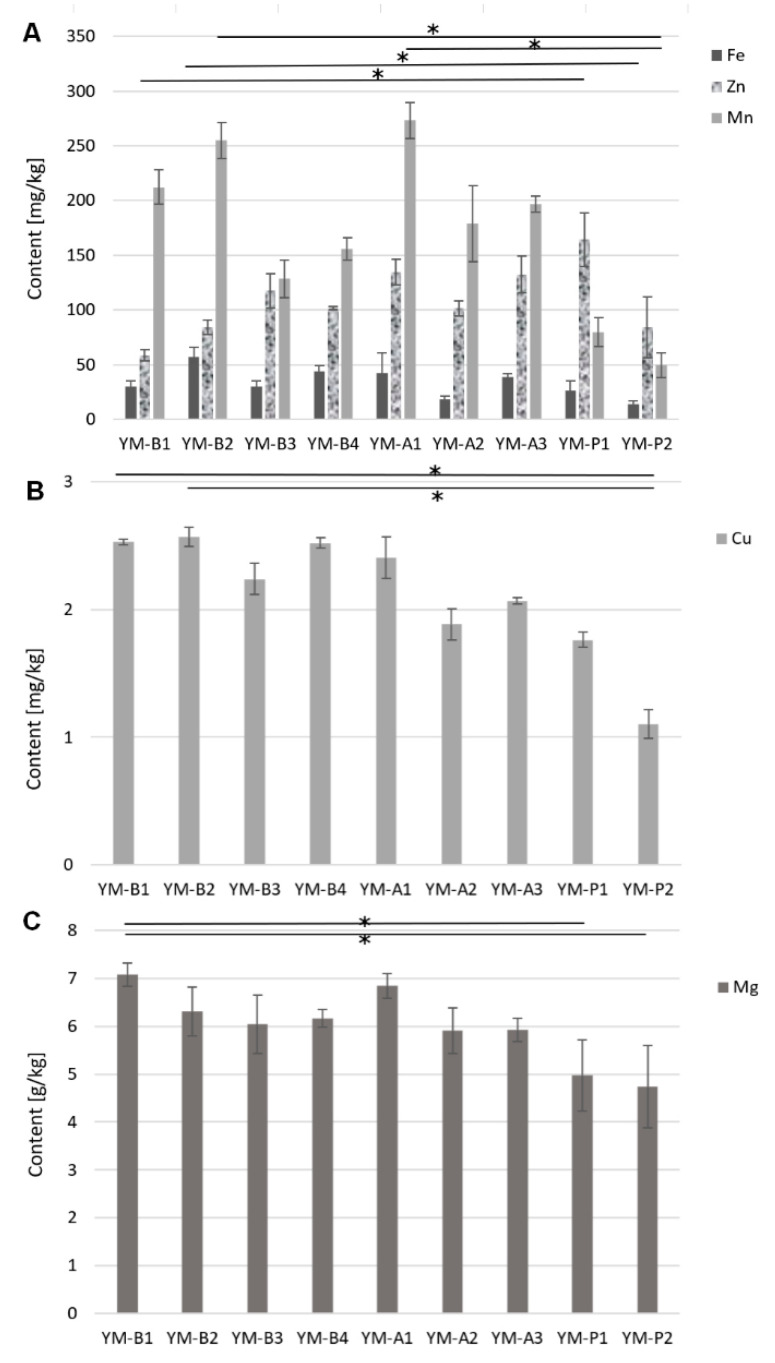
Comparison of the contents of (**A**) Fe, Zn, and Mn; (**B**) Cu; (**C**) Mg in yerba mate samples (Kruskal–Wallis test with Dunn’s post hoc test (*p* < 0.05)). Bars with an asterisk (*) are significantly different (*p* < 0.05).

Presently, there is an increasing awareness among people with regard to the impact of food and its components on human health. Studies on the content of bioactive compounds in foods can be used in the rational planning of daily diets. Bioelements have many functions in the human body, and therefore, it is necessary to provide them through food. Figure 2 shows the contents of the studied elements in individual samples.

On the basis of the obtained results for the contents of bioelements in dried yerba mate and considering the Recommended Dietary Allowance (RDA) of the examined bioelements, dried samples with the highest and lowest contents of particular elements were selected for further analyses. Table 1 shows the results of the determination of the selected metal ions in the prepared yerba mate infusions. Next, we assessed the amount of the determined elements extracted from the dried fruit into the infusion and the extent to which these elements covered the RDA for humans, and the results are presented in Table 2. Knowledge of the elemental composition of a given product allows the assessment of the products that can prevent deficiencies in macro- and microelements.

Several studies have investigated the elemental content of holly leaves. The contents of Mg, Ca, K, P, Cu, Zn, Mn, Fe, Na, Al, Cd, and Pb were found to vary significantly in available sources [27,28,29,30]. In the present study, the concentrations of Mg in the infusion from samples YM-B1 and YM-P2 were 144.8 mg/L and 44.5 mg/L, respectively. The RDAs for Mg are 420 mg and 320 mg for adult men and women, respectively [31]. Mg deficiency is becoming more common among people, and hence, Mg supplementation is recommended. Early symptoms that accompany Mg deficits include loss of appetite, chronic fatigue, and vomiting. By contrast, severe hypomagnesemia manifests as increased neuromuscular excitability in the form of muscle tremors, wrist cramps, and muscle spasms. Disturbances in heart rhythm, such as atrial and ventricular tachycardia, also occur. Mg deficiency very often accompanies deficiencies of other minerals, such as Ca or K [32].

The concentrations of Zn in the infusion from samples YM-P1 and YM-B1 were 3.14 mg/L and 1.00 mg/L, respectively. The RDAs for Zn are 11 mg and 8 mg for adult men and women, respectively [33]. Long-term intake of Zn ions from food in amounts lower than the recommended intake can lead to many diseases. In children and adolescents, these disorders include poor growth and delayed development. Cellular response mechanisms in patients with Zn deficiency are not as enhanced as those of patients with normal blood Zn levels. Numerous skin lesions appear, which become worse with increasing deficiency of Zn. Wound healing becomes more difficult. The condition of the hair and nails deteriorates, leading to increased brittleness and a tendency for hair loss. In more severe cases, the lingual papillae may disappear [34].

The concentrations of Mn in infusions from samples YM-A1 and YM-P2 were 3.67 mg/L and 1.70 mg/L, respectively. Drinking 1 L per day of infusions prepared using the method described in this study can cover 34.5% of daily Mg requirements for men and 45.2% of those for women; 28.6% of Zn requirements for men and 39.3% of those for women; and 159.8% of Mn requirements for men and 204.1% of those for women. This implies that the amount of Mn in 1 L of the infusion exceeds the daily requirement of the human body for this element. The RDAs for Mn are 2.3 mg and 1.8 mg for adult men and women, respectively [35]. Because of the significant presence of Mn in the diet, Mn deficiencies are very rarely found. Long-term supply of Mn ions to the human body in amounts higher than the recommended amount causes symptoms similar to those of Parkinson’s disease. Although epidemiological studies are lacking, intravenous injection of Mn has been shown to decrease heart rate and blood pressure and increase P-R and QRS intervals in electrocardiography [36].

### 3.2. Organic Compounds in the Analyzed Yerba Mate Materials

Compounds found in plant materials with a protective effect against diseases caused by free radicals are ranked in the following order according to their antioxidant activity: phenolic acids > flavonoids > ascorbic acid > tocopherols > purines. In the analyzed material, the following organic compounds with significant antioxidant activity were detected: phenolic acids, flavonoids, and caffeine. 

According to the macroscopic analysis of the samples, the most powdered one was YM-B1, and it had the highest content of organic compounds. Similar content was noted in yerba mate samples from Brazil (YM-B2 and YM-B4). An important point to note is that these samples contained mainly leaf blades and differed from the Argentina and Uruguay samples because stems formed a major part of this sample. Compared with the leaf blade, stems contain more cellulose than organic compounds. The lowest content of organic compounds was found in the roasted material (YM-B3, roasted), which was related to the degradation of these compounds by thermal processing (Table 3). Industrial processing could affect the polyphenol content and their composition, as well as the antioxidant activity of Yerba Mate extracts [37].

Among the organic compounds, the highest levels were detected for neochlorogenic acid, chlorogenic acid, and cryptochlorogenic acid, with the highest differences in the amount observed for the last compound. The level of the quantitatively dominant neochlorogenic acid ranged from 1.24 mg/g d.w. to 39.03 mg/g d.w. The highest content of phenolic compounds was determined in the three samples from Brazil, except for one sample that underwent thermal processing by roasting (YM-B3). In the roasted product, similar concentrations of all organic compounds were detected.

Among flavonoids, rutoside and astragalin were detected. The amount of astragalin ranged from 0.14 to 1.61 mg/g d.w., whereas that of rutoside ranged from 0.27 to 8.77 mg/g d.w. (YM-B1).

The highest levels of phenolic compounds were detected in the leaf extract. The total phenolic content (TPC) obtained from *I. paraguariensis* was 51 mg/g d.w. The content of 46 different polyphenols was also quantitatively determined, among which hydroxycinnamic acid derivatives were the predominant group. The dominant compounds were 3-caffeoylquinic acid (chlorogenic acid; 26.8–28.8%), 5-caffeoylquinic acid (neochlorogenic acid; 21.1–22.4%), 4-caffeoylquinic acid (cryptochlorogenic acid; 1.6–14.2%), 3,5-dicaoylquinic acid (9.5–11.3%), and rutin (7.1–7.8%) [6]. 

The alkaloids found in the leaves of *I. paraguariensis* are purine derivatives, known as purine alkaloids, for example, caffeine. In the present study, the content of caffeine in the prepared infusions ranged from 0.18 to 1.17 mg/g d.w. Similar to the results for phenolic compounds, the highest caffeine content was determined in yerba mate preparations from Brazil, except for the roasted product. 

The caffeine content commonly found in dried yerba mate is 1–2%, and that of theobromine is 0.3–0.9% of dry matter. Compared with caffeine and theobromine, theophylline is present in a very small amount in the leaves of yerba mate, and its content in one cup of infusion of this product (about 150 mL) is approximately 78 mg, which is very similar to the caffeine content in coffee (approximately 85 mg). However, it should be noted that in traditional yerba mate brewing, the volume of the beverage consumed sometimes reaches even 500 mL, and this amount of the beverage contains approximately 260 mg or more of caffeine [38].

Previous studies have shown that the mean intake of polyphenols in the diet was 1756.5 ± 695.8 mg/d (median = 1662.5 mg/d) for individuals from an urban population of Krakow, Poland. The highest intake was of isomers of chlorogenic acid, which largely originated from coffee [39]. The average intake of total phenolic compounds by the Brazilian population was 460.15 mg/day, which was mainly derived from beverages (48.9%), especially coffee and legumes (19.5%) [40]. Carnauba et al. reported that nonalcoholic beverages and fruits were the major sources of polyphenols in the Brazilian population, while coffee and orange juice were the main contributors to polyphenol intake [41]. Tea and coffee are the caffeinated beverages commonly consumed worldwide. Yerba mate is another caffeinated drink that is becoming increasingly popular [6]. Coffee, tea, and yerba mate beverages, because of their widespread consumption, can therefore be an important source of compounds with antioxidant properties in the daily diet [42].

The comparison of the content of polyphenols in Yerba Mate is difficult because of the different methods used to separate them from the tested raw material. The authors not only used water infusions prepared at different temperatures and times to simulate and reflect the process carried out by consumers, but they also carried out the extraction process continuously for 4 h in a Soxhlet apparatus, both with water and organic solvents, such as ethanol or ethyl ether, at 76 °C, 40 °C, and 97 °C or triplicate extracted with 2 N hydrochloric acid in aqueous methanol (50:50, *v/v*) for 1 h by constant shaking at room temperature [43,44,45].

The analysis of non-hallucinogenic indole derivatives confirmed the presence of trace amounts of L-tryptophan and 5-hyroxy-L-tryptophan in the extracts of YM-B1 and YM-B2 (Brazil). 5-Hydroxytryptophan is a compound produced in the human body from the amino acid L-tryptophan. It is a precursor of the neurotransmitter serotonin and the hormone melatonin. L-Tryptophan and 5-hydroxy-L-tryptophan have a sleep-depriving effect and support the treatment of depression. They are also precursors of serotonin and melatonin—endogenous compounds responsible for regulating the circadian cycle of the human body. Serotonin also has antioxidant and anticancer properties.

### 3.3. Antioxidant Activity

The antioxidant activity of infusions is shown in Table 4. The antioxidant activity was highest for the infusion prepared from the yerba mate samples from Brazil and Paraguay (YM-B1 and YM-P2) using the DPPH test. For FRAP antioxidant activity, the test sample from Brazil (YM-B4) was the highest. Thermal treatment (roasting) deprives yerba mate of organic compounds with antioxidant activity and affects the antioxidant properties of infusions prepared from these products. In the DPPH and FRAP tests used, the antioxidant activity of the infusion obtained from a sample from Brazil (YM-B3) was the lowest. Bastos et al. demonstrated that ethanolic and aqueous extracts from green yerba mate and roasted yerba mate showed excellent DPPH scavenging activity (>89%), but continuous extraction was performed for 4 h in a Soxhlet apparatus with ethanol or deionized water (100 mL) at 76 °C, 40 °C, and 97 °C, respectively. The ethanolic extracts from yerba mate, both roasted and green, with phenolic compound contents (3.80 and 2.83 mg/mL) presented high antioxidant activity, even at very low phenolic concentrations [45].

## 4. Conclusions

In the present study, the content of biologically active compounds showing antioxidant activity and selected bioelements in the infusions of nine types of traditional yerba mate available in Poland was determined. The study showed that the yerba mate samples from Brazil were the richest in bioelements, while the samples from Paraguay showed the lowest content of these bioelements. By drinking 1 L of the infusion prepared according to the method described in this study, one can cover 34.5% of the daily requirement of Mg for men and 45.2% of that for women, along with 28.6% of the daily requirement of Zn for men and 39.3% of that for women, whereas for Mn, the amount exceeds the daily requirement for this element. It is generally accepted that phenolic compounds, especially chlorogenic acid and its derivatives for which yerba mate can be a valuable dietary source, are mainly responsible for alleviating oxidative stress. It has been shown that infusions, especially those made from raw materials containing mainly leaf blades, are a good source of phenolic compounds and caffeine, i.e., compounds exhibiting antioxidant properties. In conclusion, it should be emphasized that thermal processing (roasting) deprives yerba mate of organic compounds with antioxidant activity. Therefore, to better preserve the content and stability of beneficial antioxidants in yerba mate, it is essential to consider an appropriate method to prepare these products.

## Figures and Tables

**Table 1:** Mg(II), Zn(II), and Mn(II) contents of yerba mate infusions.

Element	Sample	Content (mg/L)	% of Content in Infusions Compared to the Content in Dried Material
Mg	YM-B1	144.75 ± 6.8	29.9%
	YM-P2	44.45 ± 13.7	14.0%
Zn	YM-P1	3.14 ± 0.0	27.8%
	YM-B1	1.00 ± 0.0	24.9%
Mn	YM-A1	3.67 ± 0.1	18.9%
	YM-P2	1.70 ± 0.0	50.2%

**Table 2:** Coverage of the daily recommended requirement when drinking 1 L of yerba mate infusion per day prepared using the described method.

Element	Sample	% RDA at Intake of Approximately 1 L per Day	
		Men	Women
Mg	YM-B1	34.5%	45.2%
	YM-P2	10.6%	13.9%
Zn	YM-P1	28.6%	39.3%
	YM-B1	9.1%	12.5%
Mn	YM-A1	159.8%	204.1%
	YM-P2	80.0%	94.5%

**Table 3:** Content of organic compounds in yerba mate samples analyzed in the present research.

	Neochlorogenic Acid	Chlorogenic Acid	Cryptochlorogenic Acid	Caffeic Acid	4-Feruloylquinic Acid	isochlorogenic Acid	Rutoside	Astragalin	Caffeine
[mg/g dry mass] ± SD									
YM-B1	39.03 ± 0.64 ^a^	19.00 ± 0.29 ^a^	17.84 ± 0.29 ^a^	0.60 ± 0.02 ^a^	2.92 ± 0.10 ^a^	28.82 ± 0.47 ^a^	8.77 ± 0.26 ^a^	1.61 ± 1.10	1.17 ± 0.00 ^a^
YM-B2	24.46 ± 0.18	12.37 ± 0.06	12.18 ± 0.19	0.44 ± 0.01	2.20 ± 0.02	22.20 ± 0.20	6.53 ± 0.09	1.61 ± 0.01	0.74 ± 0.02
YM-B4	25.95 ± 0.20 ^c^	12.12 ± 0.30	13.59 ± 0.30	0.39 ± 0.02	1.75 ± 0.08	24.32 ± 0.20	8.19 ± 0.23	1.40 ± 0.03	0.86 ± 0.02 ^b^
YM-A1	15.61 ± 0.28	7.04 ± 0.24	6.91 ± 0.36	0.21 ± 0.01	1.19 ± 0.08	11.26 ± 0.32	4.98 ± 0.21	0.93 ± 0.03	0.42 ± 0.01
YM-A2	20.06 ± 0.05	10.22 ± 0.24	11.60 ± 0.33	0.34 ± 0.03	1.40 ± 0.05	14.83 ± 0.19	6.18 ± 0.14	0.94 ± 0.01	0.59 ± 0.01
YM-A3	23.81 ± 0.28	12.13 ± 0.28	12.07 ± 0.42	0.26 ± 0.01	1.52 ± 0.12	21.06 ± 0.56	6.51 ± 0.49	1.00 ± 0.03	0.70 ± 0.01
YM-P1	16.86 ± 0.07	10.52 ± 0.36	11.36 ± 0.48	0.38 ± 0.02	1.91 ± 0.15	15.90 ± 0.12	5.51 ± 0.16	0.98 ± 0.28	0.46 ± 0.02
YM-P2	5.39 ± 0.09 ^a^	3.21 ± 0.06 ^a^	3.48 ± 0.10 ^a^	0.07 ± 0.00 ^a^	0.57 ± 0.00 ^a^	4.78 ± 0.13 ^a^	1.73 ± 0.03 ^a^	0.38 ± 0.01	0.18 ± 0.00 ^ab^
YM-B3 *	1.24 ± 0.03	1.77 ± 0.050	1.32 ± 0.01	0.05 ± 0.00	0.56 ± 0.02	0.45 ± 0.02	0.27 ± 0.01	0.14 ± 0.00	0.29 ± 0.01

Each analysis was performed in triplicate (Kruskal–Wallis test with Dunn’s post hoc test; values followed by a different letter (a, b, c) within the same row are significantly different (*p* < 0.05) * roasted yerba mate (not statistically analyzed because roasting degrades organic compounds).

**Table 4:** Comparison of the total phenolic compounds and antioxidant activity of analyze yerba mate samples.

	DPPH° *	FRAP **	TPC ***	TFC ***
YM-B1	61.29 ± 1.44 ^e^	69.39 ± 2.84 ^c^	86.23 ± 2.84 ^c^	475.56 ± 11.74 ^d^
YM-B2	52.34 ± 1.98 ^cd^	42.04 ± 1.86 ^b^	70.16 ± 2.61 ^c^	338.35 ± 33.84 ^bc^
YM-B3	20.20 ± 0.77 ^a^	1.94 ± 0.49 ^a^	22.05 ± 0.52 ^a^	36.65 ± 2.82 ^a^
YM-B4	46.23 ± 1.38 ^bc^	117.88 ± 14.45 ^e^	62.92 ± 2.03 ^bc^	360.91 ± 14.92 ^bc^
YM-A1	53.03 ± 0.81 ^d^	62.75 ± 1.77 ^c^	82.23 ± 3.05 ^c^	582.71 ± 24.43 ^e^
YM-A2	43.46 ± 1.27 ^b^	52.22 ± 3.87 ^bc^	57.74 ± 3.88 ^b^	340.23 ± 21.35 ^bc^
YM-A3	50.95 ± 3.01 ^cd^	56.87 ± 1.67 ^bc^	66.03 ± 3.81 ^bc^	329.89 ± 28.19 ^b^
YM-P1	41.86 ± 2.49 ^b^	41.77 ± 3.98 ^b^	56.35 ± 2.01 ^b^	332.71 ± 25.84 ^b^
YM-P2	59.55 ± 1.61 ^e^	62.15 ± 3.83 ^c^	85.06 ± 5.97 ^c^	404.13 ± 14.19 ^c^

* % of a reduced radical DPPH°, ** µM Fe^2+^ per 1 mL of Yerba Mate infusion, *** mg per 1 mL of Yerba Mate infusion. Each analysis was performed in triplicate (Tukey test: values followed by a different letter (a, b, c, d, e) within the same row are significantly different (*p* < 0.05).

## Data Availability

Data is contained within the article.
